# The effectiveness of stress management training given to first‐class health major students in perceiving and coping with stress and developing resilience: A randomized controlled trial

**DOI:** 10.1111/aphw.70014

**Published:** 2025-03-05

**Authors:** Sercan Mansuroğlu

**Affiliations:** ^1^ Department of Health Care Services Kütahya Health Sciences University Kütahya Vocational School Kütahya Turkey

**Keywords:** health major students, mental health, perceived stress, psychological resilience, stress management

## Abstract

Stress management plays a vital role in protecting students' mental health. Effective stress management helps them to recognize sources of stress and cope with the problems caused by stress, helping them to become more mentally resilient in the long run. This study was a pretest–posttest randomized controlled psychoeducational intervention in which the effect of stress management training given to first‐class health major students on their perceived stress, coping methods and psychological resilience was examined. The study was conducted with 102 associate degree first‐class health major students, 51 intervention, and 51 control. Intervention group received stress management training consisting of seven modules for 7 weeks. No intervention was given to the control group. Data were collected using sociodemographic form, perceived stress scale, stress coping methods scale, and short psychological resilience scale. Pre‐test and post‐tests were administered to both groups. In the analysis of within‐group differences of the intervention group, there was a significant decrease in perceived stress scores and a significant increase in coping methods and psychological resilience scores. Between the groups, there was a significant difference in the post‐test scores of the intervention group compared to the control group. In the Covariance analysis in which the pre‐test effect was controlled, it was determined that the effect of the stress management training on the perceived stress level of the students was 22.1% (large effect), the effect on coping methods with stress was 5.1% (medium effect), and the effect on psychological resilience was 22.6% (large effect) in favor of the intervention group. As a result, stress management training given to health major students decreased their perceived stress levels, improved their coping methods with stress, and increased psychological resilience levels.

## INTRODUCTION

Perceived stress refers to the level of stress felt by the individual in relation to the situations in his/her life. Under the influence of personal experiences, familial characteristics, education and training status, values, and belief systems, each individual perceives the event encountered differently and attributes different meanings. The more negative the individual's feelings and thoughts about the situations, the higher the perceived stress level (Fauci et al., 2023). The stress response does not depend on what happens in the environment, but on how the individual reacts to what happens. For this reason, stress emerges from the interaction of the individual and the events (Alwhaibi et al., 2023).

Determining the factors that trigger perceived stress in university students is important in terms of preventing mental, physical, and behavioral problems related to stress. There are many factors that directly and indirectly affect the process of exposure to, reaction to, and coping with stressful events in young people. University life, which is one of the most important processes before entering professional life, offers new social environments where young people can discover different perspectives and lifestyles. Young people who see and try many new social roles in university life try to establish a balance between their internal expectations and environmental expectations (Özavci et al., 2023). This period, when new expectations emerge, brings along some difficulties that may increase students' stress levels. Adaptation problems that students may encounter in university life may be problems experienced during the registration process, communication with friends, staying away from the family if the university education will continue in a different city, housing problems, and financial problems. Young people who lack the qualities that will enable them to cope effectively with these difficulties perceive stress more intensely, which can negatively affect their interpersonal relationships, harmony, and success and cause them to experience various mental problems (Alwhaibi et al., 2023; Marendić et al., 2024).

When faced with stressful situations that may lead to the emergence of mental problems, it is seen that some people's negative mood persists for a long time, while some people can get out of this mood in a short time and return to their normal lives. One of the possible explanations for this situation is the differences in psychological resilience levels. The views on stress, stress‐inducing situations, and getting stronger as they cope with these difficulties come together around the concept of psychological resilience in various fields (Litwic‐Kaminska et al., 2023; Martínez‐Rubio et al., 2023).

The fact that the phenomenon of psychological resilience is a dynamic process, includes developable qualities, effective coping, and healthy adaptation processes with trauma or difficult life conditions (Southwick et al., 2014), the individual is exposed to risk or difficulty for the development of psychological resilience and achieves success in different areas of his/her life by adapting to the situation, and at the same time, individuals have various personality traits that are described as protective factors for psychological resilience two basic factors are emphasized in the concept of psychological resilience (Sisto et al., 2019). The first of these is the ability to focus on recovering from stressful life events and to quickly reach balance and recover in order to return to the starting point in a healthy way, and the second is sustainability. Sustainability can be explained as the ability to maintain similarly healthy responses in other stress situations as a result of healthy responses to stressful life events (Litwic‐Kaminska et al., 2023; Sahin‐Bayindir & Buzlu, 2019). It is seen that students who have just started their university education try to cope with issues such as making new friends, trying to adapt to a new environment, and getting used to living separately from their families (Gao et al., 2024); in the later years of their education, students try to fulfill the expectation of “being successful” expected from them, and students at the graduation stage experience stress about exams to be taken after graduation, career planning, and job placement (Gülnar et al., 2024).

Today, psychoeducation is an important component of a comprehensive treatment approach for all mental illnesses. It involves teaching disorder‐specific knowledge and priorities in both curative and preventive contexts. Psychoeducation is based on the principle that knowledge about mental illness and its causes and effects can influence people's behavior, especially when faced with stressors (Günaydin, 2022). Wyatt et al. (2017) stated that first‐year students have higher rates of self‐harm and seriously consider suicide, and that the first year at university is the most appropriate time to raise awareness and develop strategies to prevent mental problems. The intensity of the stress experienced by university students and the diversity of their problems lead them to be considered as a risk group (Zou et al., 2024), and this research, which examines the perceived stress, coping methods with stress, and psychological resilience of people in the risk group, is important in terms of providing data and giving direction to the studies to be conducted for these people. It has also been reported that stress management training is a successful intervention for university students to reduce stress and other negative mental symptoms and increase psychological well‐being (Ahmad et al., 2022; Gülnar et al., 2024; Recabarren et al., 2019; Van Daele et al., 2012; Weiss et al., 2024).

It is known that students studying in health departments experience more stress than students in other departments (McConville et al., 2017). In the related studies, it is seen that students studying in undergraduate departments related to health are mostly studied (Aloufi et al., 2021; Graves et al., 2021; Morales‐Rodríguez, 2021; Regehr et al., 2013). Although there are more studies with undergraduate students, undergraduate and associate degree health major students share the same working environment when they graduate. However, each member of the multidisciplinary team is valuable and in communication and interaction with each other (Bozkurt & Şener, 2013). Therefore, there may be mutual influence on stressful situations (Rosen et al., 2018). In the country where the study was conducted, health‐related associate degree programs have a 2‐year education period and they are educated under the name of the vocational school (Gayef, 2017). Therefore, they start working life at an earlier age compared to undergraduate education (Bozkurt & Şener, 2013). This may lead them to feel inadequate in acquiring professional knowledge and skills. This causes them to experience more adaptation problems in their professional life (Kayaalp, 2018). It is stated that because those who study in applied fields such as health sciences experience higher stress compared to other groups, studies are often conducted for these groups and they benefit more from the practices (Veena & Shastri, 2016; Yusufov et al., 2019). This situation shows that there is a need to work with associate degree students. Therefore, it is important to examine the effectiveness of stress management training given to students studying in associate degree programs of health‐related departments.

When the literature is examined, it is seen that interventions for students to reduce stress and increase resilience are encouraged (Delany et al., 2015; Regehr et al., 2013; Shiralkar et al., 2013). It is stated that intervention programs prepared especially based on awareness become easier to be used by students in daily life (Galante et al., 2018; Kiken et al., 2015). In this study, the effects of stress management training given to health major students on their perceived stress, coping methods with stress, and psychological resilience were examined. It is thought that this study will contribute to the literature and is important in terms of providing information for preventive studies to be organized for university students in the risk group, which can be considered as the future of the mental health of the society, and being an example for other psychoeducational studies to be conducted to provide stress management and strengthen the mental health of students.

### Research hypotheses

Perceived stress post‐test scores of first‐class associate degree health major students who received stress management training will be lower than those who did not receive the same training.

Methods of coping with stress post‐test scores of the first‐class associate degree health major students who received stress management training will be higher than the post‐test scores of the students who did not receive the same training.

First‐class associate degree health major students who receive stress management training will have higher psychological resilience post‐test scores than students who do not receive the same training.

## METHODS

### Setting and time

This study was conducted between March and May 2024 with first‐class students studying in the associate degree health departments of the central vocational school of a health university in Türkiye.

### Population and sample

The population of the study consisted of 126 first‐class health major students studying in the associate degree departments of the central vocational school of a health university in Türkiye. The sample consisted of students who met the inclusion criteria and voluntarily accepted to participate in the study. The sample size was calculated by performing a power analysis before the study started. Using Cohen's standardized effect size for the study, the minimum sample size was calculated as 51 for each group with a medium effect size (0.5), alpha value of 5%, and theoretical power of 80% (Cohen, 1988, 1990; Lai, 2021; Varol et al., 2015). The study was completed with 102 students, 51 intervention, and 51 control.

### Participants

One hundred and two health major students (51 intervention and 51 control) were included or excluded according to the following criteria:

***Inclusion criteria:***

- 18 years of age or older
- Being first‐class health major students studying in the associate degree departments
- Voluntary acceptance to participate in the study
- Continuing education

***Exclusion criteria:***

- Under 18 years of age
- Being associate degree second‐class health major students or Bachelor's degree or higher
- Refusing to participate in the study at any stage
- Not continuing their education

**FIGURE 1:**
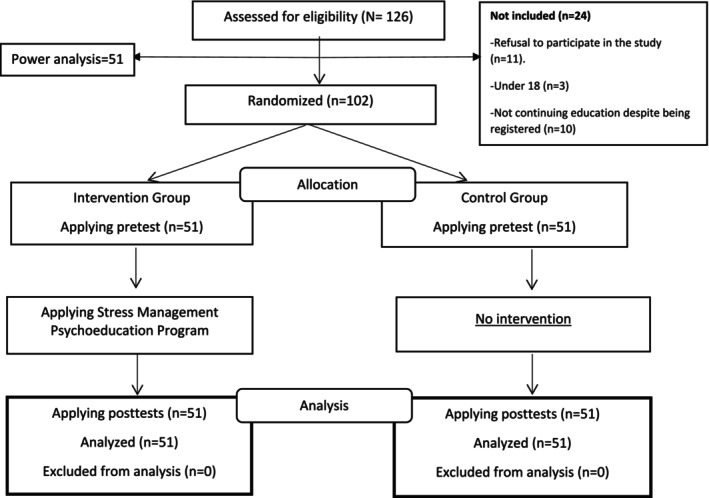
CONSORT flow diagram.

Students who did not meet the specified inclusion criteria were excluded from the study. Figure 1 shows the design of the study (CONSORT scheme). The students participating in the study were not informed which group they were in. Single blinding method was used in the study because the researcher was involved in group allocation, intervention, and data collection tasks.

### Randomization

A total of 126 first‐year students studying at a vocational school were evaluated by the researcher according to the inclusion or exclusion criteria. As a result, 102 participants were randomized and assigned to the intervention and control groups. In order to reduce selection bias in determining the intervention and control groups, the individuals to be included in the sample were determined using the www.random.org website.

### Variables of the study

The dependent variables of the study are perceived stress scale, coping methods with stress scale, and short psychological resilience scale. Stress management training is the independent variable. Sociodemographic data of the students were the control variable of the study.

### Data collection tools

The research data were collected with a form including sociodemographic characteristics of the students, Perceived Stress Scale, Stress Coping Methods Scale, and Brief Resilience Scale.

#### Sociodemographic data form

It is a form consisting of 18 questions inquiring the characteristics of the students such as age, gender, the department they study, smoking status, income status, and the people they live with (Gülnar et al., 2024; Selin & Yıldız, 2023).

#### Perceived stress scale (PSS‐14)

Developed by Cohen et al. (1983) and adapted into Turkish by Eskin et al. (2013), the scale consists of a total of 14 items and evaluates the stress perceptions of individuals in the face of certain situations encountered in their lives. The scale has a 5‐point Likert scale. The scale has two sub‐dimensions. The lowest score that can be obtained from PSS‐14 is 0, and the highest score is 56. The higher the score obtained by the individuals from the scale, the higher the stress perception. Cronbach's alpha coefficient of PSS‐14 was found to be 0.84 (Eskin et al., 2013). In this study, the total score of the scale was used, and Cronbach's alpha coefficient was calculated as 0.88 in the pre‐test and 0.85 in the post‐test for the intervention group and 0.82 in the pre‐test and 0.77 in the post‐test for the control group.

#### Stress coping methods scale (SCMS)

The coping methods scale developed by Moos (1993) and adapted into Turkish by Ballı and Kılıç (2016) was used to measure coping methods with stress. The scale consists of a five‐factor structure. Consisting of 24 statements, the scale is graded in 5‐point Likert type and the answer options are 1 ‐ *Never*, 2 ‐ *Rarely*, 3 ‐ *Sometimes*, 4 ‐ *Mostly*, and 5 ‐ *Always*. The Cronbach alpha coefficient of the scale was calculated as 0.93 (Ballı & Kılıç, 2016). In this study, the total score of the scale was used, and Cronbach's alpha coefficient was calculated as 0.81 in the pre‐test and 0.83 in the post‐test for the intervention group and 0.83 in the pre‐test and 0.75 in the post‐test for the control group.

#### Brief resilience scale (BRS)

The scale is a 5‐point Likert type, 6‐item, self‐report measurement tool developed by Smith et al. (2008) and adapted into Turkish by Doğan (2015) in order to measure the psychological resilience of individuals. After the reverse coded items in the scale are translated, high scores indicate high psychological resilience. It was reported that the Cronbach's alpha coefficient of the scale was calculated as 0.83 (Doğan, 2015). In this study, Cronbach's alpha coefficient was calculated as 0.84 in the pre‐test and 0.89 in the post‐test for the intervention group and 0.87 in the pre‐test and 0.87 in the post‐test for the control group.

### Intervention program

The stress management training, which is the intervention program of this study, is based on an innovative approach that is not only limited to the transfer of theoretical knowledge but also focuses on developing practical and effective skills that students can easily apply in their daily lives. The training enabled individuals to better recognize their sources of stress, while using emotion‐focused, appraisal‐oriented, and constructive approaches to cope with these sources (Throner et al., 2023; Weiss et al., 2024) and scientifically proven methods such as problem solving (Şenocak & Demirkıran, 2023; Yüksel, 2020), crisis management (Kiessling, 2021), anger management (Nalbatoğlu Buluş & Allahverdi, 2024), time management (Rith‐Najarian et al., 2019), and relaxation techniques (Leahy & Holland, 2009; Sertel‐Berk, 2020; Shapiro, 2017; Yusufov et al., 2019). In particular, a flexible structure was offered to adapt to the individual needs of the students, and each participant was encouraged to actively contribute to the training based on their own experiences.

The participant‐oriented nature of the training enabled students to be part of the process rather than passive listeners, which facilitated the internalization of the skills learned. Moreover, the interactive activities enriched the learning experience and made it more effective. As a result, in the short term, the training helped students recognize stress, increase their capacity to cope with stress, and increase their psychological resilience; in the long term, it can be stated that it will contribute to their preparedness for the potential stressors they may be exposed to and their consequences. These features made the training more powerful, comprehensive, and innovative than previous interventions in terms of both content and impact. The detailed content of the “stress management training,” the intervention program implemented in the study, is given in Table 1. Stress management training was given by the researcher, who has a master's degree in Community Mental Health Nursing and a doctoral degree in Mental Health and Psychiatric Nursing and has received training on stress and coping with stress.

**TABLE 1:** Stress management training content.

Modules	Contents	Methods used in training
First meeting: Explanation of the purpose of the research and the process, and application of pre‐tests		
1. Module: stress and its components	Definition and types of stress Theoretical approaches to stress Symptoms and consequences of stress Mechanism of stress formation	Theoretical expression
2. Module: stress management	The concept of stress management (individual and organizational methods) Stress management with evaluation‐oriented approaches (e.g., rational thinking, positive reinterpretation, use of humor) Stress management with problem‐oriented approaches (e.g. problem solving, time management, seeking social support, self‐control) Stress management with emotion‐oriented approaches (e.g., emotion management, exercise, meditation, and systematic relaxation)	Theoretical expression Practical exercises Reinforcement with real‐life examples Homework assignment
3. Module: crisis and its components	Definition and types of crisis Factors that cause the crisis Consequences of the crisis Crisis process (e.g., failure to recognize internal and external changes, failure to act, wrong decisions and actions, crisis period, resolution, or collapse)	Theoretical expression
4. Module: crisis management	Purposes of crisis management Importance of planning against crisis Crisis management approaches and models (e.g., receiving crisis signals, crisis preparedness and protection, crisis containment, return to normalcy, prevention, and evaluation)	Theoretical expression Reinforcement with real‐life examples Homework assignment
5. Module: anger and its components	Definition of anger and its examination in the dimensions of emotion‐thought‐behavior‐communication Theoretical approaches to anger, causes, functions, symptoms types, forms of expression, and consequences of anger	Theoretical expression
6. Module: anger management	Anger management and control (e.g., accepting, understanding, finding the source, and dealing with anger) Anger management strategies (e.g., relaxation techniques [e.g., breathing and progressive muscle exercises], cognitive restructuring, problem solving, communication enhancement, use of humor, and management of environmental factors)	Theoretical expression Practical exercises Reinforcement with real‐life examples Homework assignment
7. Module: psychological resilience	Definition of psychological resilience Challenging situations (daily evolving, suddenly evolving) Developmental processes and capacity (e.g., functioning of the system, maintaining functionality, and achieving gains) Positive psychological outcomes, risk factors, and components of psychological resilience in the individual (personal, environmental, and relational) Equipment that increases psychological resilience (e.g., intelligence, behavior, positive emotions, humor, self‐control, self‐awareness and self‐appraisal, organizing the environment, goals and ideals, and personal development)	Theoretical expression Reinforcement with real‐life examples Homework assignment

*Note*: Final meeting: Evaluation of the sessions and administration of post‐tests.

### Research procedure

In this face‐to‐face study, students who met the inclusion criteria were randomized and assigned to the groups. In the first interview with the students assigned to the intervention and control groups, students were informed about the purpose of the study and the process, and pre‐tests were applied to the students after their consent was obtained. After 1 week, students randomly assigned to the intervention group were given stress management training consisting of seven modules, each lasting 120 min (with a break), on a day determined during the week for 7 weeks. Two weeks after the training was completed, post‐tests were applied to the intervention and control group students (Table 1).

During this process, no intervention was applied to the control group. After the study was completed, the same stress management training was given to the control group.

### Ethics

For the scales used in the study, permission was obtained from the authors who developed the scales and conducted the adaptation studies into Turkish. Written permission was obtained from the ethics committee of the university where the study was conducted (number 2024/03‐04 on 05/03/2024) for ethical compliance and implementation. Informed consent was obtained after explaining the purpose of the study to the students participating in the study. In addition, the ethical principles of the current Helsinki Declaration were adhered to during the study. This study has been registered with a Clinical Trial Registry ID (Clinical Trials ID: NCT06502314).

### Analyzing the data

In this study, first, power analysis was performed to determine the sample size. Mean and standard deviation were used as descriptive statistics for quantitative variables determined by measurement, and number and percentage were used as descriptive statistics for qualitative variables determined by counting. Chi‐square test were used to analyze the similarity of the groups. After the normality analyses showed that the data had a normal distribution, independent sample *t*‐test and dependent sample *t*‐test were used for comparisons of pre‐test and post‐test scores within and between groups. Analysis of covariance (ANCOVA) was used to determine the effectiveness level of psychoeducation. In order to determine the effect size, Eta squared (*μ* ^2^) values were taken into consideration, and these values were interpreted by taking the values determined by Cohen (1992) as criteria (small if 0.01, medium if 0.06 and above, and large if 0.14 and above) (Pallant, 2017). Cronbach's alpha was calculated for reliability analysis. Analyses were performed with SPSS V25 and *p* < .05 was considered statistically significant in comparisons.

### Type and purpose of the research

The purpose of this randomized controlled trial examined the effect of stress management training given to first‐class associate degree health major students on students' perceived stress, coping methods with stress, and psychological resilience.

## RESULTS

Table 2 shows the findings related to the personal characteristics of the students in the intervention and control groups. In the intervention group, 78.4% were female and the average age was 19.68 ± 4.27. It was seen that 98% of the group was single, 49% of the group's income was equal to their expenses, 96.1% of the group was not working in an income‐generating job, 58.8% of the group's mother was a primary school graduate, 37.3% of the group's father was a high school graduate, 98% of the group's parents were alive, and 88.2% of the group's parents were together.

**TABLE 2:** Comparison of sociodemographic characteristics of health major students in intervention and control groups (*n* = 102).

Variables		*Group*			*Test statistics* ^a^	
		*Intervention* (*n = 51*)	*Control* (*n = 51*)	*Total* (*n = 102*)		
		*n* (*%*)	*n* (*%*)	*n* (*%*)	*ꭓ* ^2^	*p*
Gender	Female	42 (82.4)	40 (78.4)	82 (80.4)	0.70	.401
	Male	9 (17.6)	11 (21.6)	20 (19.6)		
Marital status	Married	1 (2.0)	1 (2.0)	2 (2.0)	0.02	.886
	Single	50 (98.0)	50 (98.0)	100 (98.0)		
Income status	Income<expense	13 (25.5)	19 (37.3)	32 (31.4)	1.74	.783
	Income = expense	23 (45.1)	25 (49.0)	48 (47.1)		
	Income > expense	15 (29.4)	7 (13.7)	22 (21.6)		
Working condition	Yes	2 (3.9)	3 (5.9)	5 (4.9)	0.13	.718
	No	49 (96.1)	48 (94.1)	97 (95.1)		
Mother education	Illiterate	1 (2.0)	1 (2.0)	2 (2.0)	23.80	.094
	Primary education	30 (58.8)	30 (58.8)	60 (58.8)		
	Middle school	8 (15.7)	8 (15.7)	16 (15.7)		
	High school	10 (19.6)	10 (19.6)	20 (19.6)		
	University	2 (3.9)	2 (3.9)	4 (3.9)		
Father education	Illiterate	0 (0,0)	0 (0,0)	0 (0,0)	5.99	.741
	Primary education	17 (33.3)	16 (31.4)	33 (32.4)		
	Middle school	14 (27.5)	12 (23.5)	26 (25.5)		
	High school	15 (29.4)	19 (37.3)	34 (33.3)		
	University	5 (9.8)	4 (7.8)	9 (8.8)		
Is the mother alive?	Yes	50 (98.0)	50 (98.0)	100 (98.0)	0.02	.886
	No	1 (2.0)	1 (2.0)	2 (2.0)		
Is the father alive?	Yes	50 (98.0)	45 (88.2)	95 (93.1)	0.13	.712
	No	1 (2.0)	6 (11.8)	7 (6.9)		
Parental partnership status	Together	45 (88.2)	40 (78.4)	85 (83.3)	0.09	.756
	Separated/ divorced	6 (11.8)	11 (21.6)	17 (16.7)		
Subjective stress level	Low	2 (3.9)	0 (0,0)	2 (2.0)	0.55	.756
	Middle	28 (54.9)	31 (60.8)	59 (57.8)		
	High	21 (41.2)	20 (39.2)	(41 (40.2)		
Having received stress training before	Yes	3 (5.9)	5 (9.8)	8 (7.8)	0.34	.556
	No	48 (94.1)	46 (90.2)	(94 (92.2)		
Previously receiving psychiatric/ psychological support	Yes	8 (15.7)	9 (17.6)	17 (16.7)	0.17	.677
	No	43 (84.3)	42 (82.4)	85 (83.3)		
Smoking	Yes	8 (15.7)	12 (23.5)	20 (19.6)	1,02	.310
	No	43 (84.3)	39 (76.5)	82 (80.4)		
		**Mean ± SD**	**Mean ± SD**		*t* ^b^	*p*
Age (years)		19.17 ± 2.75	19.68 ± 4.27		−0.70	.482

^a^ Chi‐square,

^b^ *t*‐test.

In the intervention group, 54.9% of the students stated that their stress levels were moderate, 94.1% had not received any training on stress management before, 84.3% had not received any psychiatric support, and 84.3% did not smoke. In the control group, 82.4% were female and the mean age was 19.17 ± 2.75 years. In the group, 98% were single, 45.1% had an income equal to their expenditures, 94.1% did not work in an income‐generating job, 58.8% of the mother and 33.3% of the father were primary school graduates, 98% of the mother and 88.2% of the father were alive, and 78.4% of the parents were together. It was determined that 60.8% of the students in the intervention group stated that their stress levels were moderate, 90.2% had not received any training on stress management before, 82.4% had not received any psychiatric support, and 76.5% did not smoke. The difference between the sociodemographic characteristics of the students in the intervention and control groups was not statistically significant, and both groups showed similar characteristics (*p* > .05) (Table 2).

In this study, in which the effect of stress management training applied to health major students was examined, it was found that the students who received the psychoeducation program showed a significant improvement compared to the pre‐test scores and compared to the control group and that the program was effective. In Table 3, the mean total scores of the students in the experimental and control groups were compared. There was no significant difference between the pre‐test scores of the participants in the intervention and control groups (*p* > .05). When the intra‐group differences of the individuals in the intervention group were analyzed, it was observed that there was a significant decrease in the post‐test scores of the PSS‐14 (*p* < .05) and a significant increase in the scores of the SCMS (*p* < .05) and BRS (*p* < .001). When the differences between the groups were analyzed, it was seen that there was a significant difference between the post‐test scores of the intervention group compared to the control group in terms of PSS‐14 (*p* < .001), SCMS (*p* < .05), and BRS (*p* < .001). In the control group, no statistically significant difference was found between the pre‐test and post‐test intra‐ and inter‐group differences of PSS‐14, SCMS, and BRS (*p* > .05) (Table 3).

**TABLE 3:** Comparison of the scores of the health major students in the intervention and control groups on the pre‐test–post‐test scales within and between groups (*n* = 102).

Scales	Tests	Group		Difference between groups^b^
		*Intervention*	*Control*	
		*X ± SD*	*X ± SD*	
		(*n = 51*)	(*n = 51*)	
PSS	Pre‐test	41.70 ± 8.46	42.54 ± 6.62	*t* = −0.560 *p* = .577
	Post‐test	36.50 ± 5.91	42.41 ± 5.75	*t* = −5.109 ***p* < .001**
	Within‐group difference^a^	*t* = 3.424 ***p* = .001**	*t* = −0.542 *p* = .590	
SCMS	Pre‐test	76.98 ± 10.39	80.47 ± 10.95	*t* = −1.651 *p* = .102
	Post‐test	85.82 ± 11.40	80.96 ± 11.28	*t* = 2.164 ***p* = .033**
	**Within‐group difference** ^a^	*t* = −3.096 ***p* = .003**	*t* = −0.237 *p* = .814	
BRS	Pre‐test	16.96 ± 5.37	16.68 ± 5.15	*t* = 0.263 *p* = .793
	Post‐test	22.15 ± 4.57	17.43 ± 4.99	*t* = 4.985 ***p* < .001**
	**Within‐group difference** ^a^	*t* = −5.192 ***p* < .001**	*t* = −1.456 *p* = .152	

*Note*: The test statistics shown in bold in the table show that the applied test gives meaningful results.

^a^ Paired samples *t*‐test.

^b^ Independent samples *t*‐test.

BRS = Brief resilience scale; PSS = Perceived stress scale; SCMS = Stress coping methods scale.

Table 4 shows the covariance analysis of the post‐test scores of the health major students in the intervention and control groups. When the effect of the pre‐tests was removed, the effect of the stress management training on the perceived stress level of the students was 22.1% (large effect) in favor of the intervention group (*F*[1,99] = 28.120; *p* < .001; *η* ^2^ = .221), the effect on coping methods with stress was 5.1% (medium effect) in favor of the intervention group (*F*[1.99] = 5.363; *p* < .05); *η* ^2^ = .051), and the effect on psychological resilience was 22.6% (large effect) in favor of the intervention group (*F*[1.99] = 28.967; *p* < .001); *η* ^2^ = .226).

**TABLE 4:** ANCOVA results of the adjusted post‐test scores of PSS, SCMS, and BRS of health major students in the intervention and control group (*n* = 102).

Scales	Source	Sum of squares	df	Mean square	*F*	Sig	Eta squared (*η* ^2^)
PSS	Pre‐test	555.923	1	555.923	19.330	.000	.163
	Group	808.727	1	808.727	28.120	.000	.221
	Error	2847.175	99	28.759			
	Total	163121.000	102				
SCMS	Pre‐test	153.952	1	153.952	1.198	.276	.012
	Group	688.990	1	688.990	5.363	.023	.051
	Error	12719.382	99	128.479			
	Total	722810.000	102				
BRS	Pre‐test	434.951	1	434.951	23.197	.000	.190
	Group	543.145	1	543.145	28.967	.000	.226
	Error	1856.303	99	18.751			
	Total	42825.000	102				

*Note*: ANCOVA = Analysis of covariance; BRS = Brief resilience scale; PSS = Perceived stress scale; SCMS = Stress coping methods scale.

## DISCUSSION

In this study, which examined the effect of stress management training given to first‐class students studying in health departments on their perceived stress, coping methods with stress, and psychological resilience, the psychoeducation had a significant and wide effect on the perceived stress levels of the students and caused the post‐test mean scores of the intervention group to be lower than the control group. Therefore, Hypothesis H1 was accepted. In studies examining the effectiveness of stress management training with university students, it was reported that stress management training given to students is an effective and important factor in reducing their stress levels (Gülnar et al., 2024; Weiss et al., 2024). Houston et al. (2017) reported that the program they implemented reduced the stress levels of university students and had a moderate effect. In addition, systematic review and meta‐analysis studies in which the evaluation of the studies conducted on university students to reduce stress are evaluated also provide evidence of the effectiveness of this training on university students in general (Ahmad et al., 2022; Van Daele et al., 2012). Furthermore, systematic analysis study examined intervention studies to reduce stress levels in nursing students and found that intervention programs were effective, and there was a decrease in stress levels (Aloufi et al., 2021). The psychoeducation given in this study was carefully prepared by taking into account the factors that play a role in the formation of stress. Therefore, it can be said that the determination that the students in the intervention group experienced a decrease in their perceived stress levels compared to the control group allowed obtaining results consistent with the literature. However, the stress management training that the students in the intervention group received compared to those in the control group had a significant and moderate effect on the methods of coping with stress, and the post‐test mean scores of the students in the intervention group were higher than those in the control group. In line with the findings of the current study, Bani Ahmad and Meriç (2021) reported that psychoeducation on stress management in their study was an effective method to reduce the stress levels of students in the intervention group and improve their coping capacity. In a study conducted with nursing students, it was found that structured group psychoeducation applied to the experimental group reduced stress and increased the use of active/problem‐focused coping in terms of effective coping with stress (Günaydin, 2022). Nguyen et al. (2023) reported that the effectiveness of the stress program applied to first‐year medical students was significant and that it greatly benefited students in developing coping strategies. Emotion‐focused coping approaches are among the most effective methods in developing strategies to cope with stress (Alotaibi et al., 2024). In another study, it was found that the intervention to improve coping skills with young people had a significant effect on the reduction of stress levels and the development of coping skills in the experimental group compared to the controls (Özkan & Altuntaş, 2024). While it is seen that the findings obtained in the current study are supported by the literature, it can be concluded that the approaches that should be applied to ensure stress management are sufficiently adopted and useful by the students. Therefore, hypothesis H2 is accepted. The development of stress coping strategies in university students can have a positive impact on students' psychological well‐being and become a protective factor to prevent mental health problems.

It is reported that university education represents an important developmental period, and young adulthood is a sensitive period in terms of the onset of common psychiatric conditions (Buizza et al., 2022). Fitzgibbon and Murphy (2023) reported in their study that the application of positive coping strategies in stress management gave health major students the ability to develop strategies to manage stress, led to a decrease in their perceived stress levels, and improved their psychological resilience. When the psychological resilience levels of the groups in this study were compared, it was seen that the psychoeducation given to the students in the intervention group had a significant and large effect by providing higher post‐test scores than the control group that did not receive the same training. Therefore, hypothesis H3 was accepted. Pehlivan Sarıbudak (2024) reported positive results in the development of psychological resilience and coping skills with stress as a result of the stress management course given to nursing students. Houston et al. (2017) reported that resilience and coping intervention had a small effect on the resilience of university students. In a study examining psychological interventions to increase resilience in health major students, it was reported that the intervention groups reported higher levels of resilience compared to controls. In the same study, it was also reported that previous interventions to increase resilience helped students cope with stress and protect themselves against negative effects on their mental health (Kunzler et al., 2020). Akhtar and Kroener‐Herwig (2019) reported that the psychological resilience of students exposed to stress who developed positive coping strategies was higher. One of the features of the stress management training given to students in this study is to help students gain awareness of stress. Awareness is an internal protective factor that regulates individuals' attention and is an important factor that increases students' psychological resilience. Low stress is closely related to psychological resilience as it ensures that the individual is in a positive mental state and attitude (Chen et al., 2021; Hughes et al., 2022; Thanoi et al., 2023). It has also been reported that psychological resilience is an important predictor of stress (Berdida et al., 2023; Klainin‐Yobas et al., 2021; Thanoi et al., 2023). Furthermore, in a systematic review of resilience effectiveness interventions, it was reported that generalized stress‐focused programs had a small to moderate effect on increasing resilience (Leppin et al., 2014). As can be seen, the concepts of stress and psychological resilience complement each other. Research supports that resilience is a skill rather than a trait and takes time to develop (Leys et al., 2020). It can be said that the 7‐week intervention program in the current study had a significant effect on developing resilience in students. In this study, it can be said that as a result of the psychoeducation received by the intervention group, their perceived stress levels decreased and their psychological resilience increased as a natural result of the development of their methods of coping with stress.

In this study, stress management training is a psychoeducation that raises awareness of students in terms of perceiving, managing, and developing resilience to stress, and it is important in terms of providing an important evidence to the literature by contributing positively to the mental health of associate degree health major students, which has been relatively less studied before.

### Limitations

It can be said that the fact that the data of the study are based on the self‐reports of the students, that it has a single‐blind design, that it is limited to associate degree students studying in the field of health, and that it can be generalized to the group at a similar level can be said to be among the limitations of the study. In addition, the lack of follow‐up in the study is another limitation. It can be thought that a similar study with university students studying at all levels of the health field (e.g., undergraduate departments such as nursing, midwifery, medicine, and dentistry and different associate degree departments) with a more diverse sample and by including follow‐up with other blinding techniques may contribute to the literature with more inclusive findings.

## CONCLUSION

In this study, it was concluded that stress management training given to first class students studying in associate degree health departments decreased their perceived stress levels and increased their coping methods and psychological resilience levels. Stress has many effects on the mental health of students at the beginning of their university education. In order to adapt and overcome the differences in their lives with a new level of education, it is necessary to perceive and cope with stressors correctly. This can be possible with a strong psychological resilience and may even make situations easier. Considering the potential stress that the intensive and exhausting pace of the health sector may cause on individuals, the acquisition of the ability to manage stress before graduation may enable individuals to have more robust relationships and interactions with themselves and with other professional members or patients/patient relatives who interact with them after graduation, thus contributing to the strengthening and maintenance of public mental health. It can be said that stress management training will meet all of these requirements. It is strongly recommended that stress management training should be given to all health‐related education levels and included in the curricula.

## CONTRIBUTION TO PRACTICE

It can be said that stress management training can help individuals recognize the symptoms of stress and gain awareness. This may enable them to understand the causes and effects of stress and learn different strategies to cope with stress. The negative effects of stress on health are obvious. This training can have a positive effect on the psychological health of individuals and help to reduce the risk of negativities that may threaten health. The gains to be obtained from stress management training can provide motivation for individuals to recognize and meet their own needs. Self‐care practices such as healthy lifestyle behaviors and sleep patterns, breathing exercises, time management techniques, problem solving skills, and emotional regulation techniques can act as reinforcers in coping with stress more effectively. As a result, this training can be considered to be effective in helping future health professionals to be aware of coping with individual and organizational stressors by enabling them to create a healthier working environment for themselves while helping to identify and manage potential sources of stress at the beginning of their professional life.

## AUTHOR CONTRIBUTIONS

**Sercan Mansuroğlu**: Concept, Design, Supervision, Resources, Materials, Data Collection and/or Processing, Analysis and/or Interpretation, Literature Search, Writing Manuscript, and Critical Review.

## CONFLICT OF INTEREST STATEMENT

The authors have nothing to report.

## ETHICS STATEMENT

Written permissions were obtained from the ethics committee of the Kütahya Health Sciences University (number 2024/03‐04 on 05/03/2024). The ethical principles of the current declaration of Helsinki were followed throughout the study.

## Data Availability

The data that support the findings of this study are available from the corresponding author, upon reasonable request.
